# Is an Energy Surplus Required to Maximize Skeletal Muscle Hypertrophy Associated With Resistance Training

**DOI:** 10.3389/fnut.2019.00131

**Published:** 2019-08-20

**Authors:** Gary John Slater, Brad P. Dieter, Damian James Marsh, Eric Russell Helms, Gregory Shaw, Juma Iraki

**Affiliations:** ^1^School of Health and Sport Sciences, University of the Sunshine Coast, Maroochydore, QLD, Australia; ^2^Australian Institute of Sport, Canberra, ACT, Australia; ^3^Department of Pharmaceutical Sciences, Washington State University, WA Spokane, WA, United States; ^4^Fiji Rugby Union, Suva, Fiji; ^5^Auckland University of Technology, Sports Performance Research Institute New Zealand, Auckland, New Zealand; ^6^Swimming Australia, Brisbane, QLD, Australia; ^7^Iraki Nutrition AS, Fjerdingby, Norway

**Keywords:** muscle hypertrophy, sports nutrition, resistance exercise, diet, nutrient timing

## Abstract

Resistance training is commonly prescribed to enhance strength/power qualities and is achieved via improved neuromuscular recruitment, fiber type transition, and/ or skeletal muscle hypertrophy. The rate and amount of muscle hypertrophy associated with resistance training is influenced by a wide array of variables including the training program, plus training experience, gender, genetic predisposition, and nutritional status of the individual. Various dietary interventions have been proposed to influence muscle hypertrophy, including manipulation of protein intake, specific supplement prescription, and creation of an energy surplus. While recent research has provided significant insight into optimization of dietary protein intake and application of evidence based supplements, the specific energy surplus required to facilitate muscle hypertrophy is unknown. However, there is clear evidence of an anabolic stimulus possible from an energy surplus, even independent of resistance training. Common textbook recommendations are often based solely on the assumed energy stored within the tissue being assimilated. Unfortunately, such guidance likely fails to account for other energetically expensive processes associated with muscle hypertrophy, the acute metabolic adjustments that occur in response to an energy surplus, or individual nuances like training experience and energy status of the individual. Given the ambiguous nature of these calculations, it is not surprising to see broad ranging guidance on energy needs. These estimates have never been validated in a resistance training population to confirm the “sweet spot” for an energy surplus that facilitates optimal rates of muscle gain relative to fat mass. This review not only addresses the influence of an energy surplus on resistance training outcomes, but also explores other pertinent issues, including “how much should energy intake be increased,” “where should this extra energy come from,” and “when should this extra energy be consumed.” Several gaps in the literature are identified, with the hope this will stimulate further research interest in this area. Having a broader appreciation of these issues will assist practitioners in the establishment of dietary strategies that facilitate resistance training adaptations while also addressing other important nutrition related issues such as optimization of fuelling and recovery goals. Practical issues like the management of satiety when attempting to increase energy intake are also addressed.

## Introduction

Resistance training is commonly prescribed to increase underlying strength and power qualities in an attempt to improve athletic performance. The enhancement of these qualities may be derived from a range of potential adaptations including improved neuromuscular recruitment, fiber type transition, and/ or skeletal muscle hypertrophy. Promoting hypertrophy is especially important in strength sports, given the strong relationship between fat free mass (FFM) and competitive lifting performance (1, 2). Furthermore, in contact sports such as rugby union, larger players have a clear advantage (3) which is highlighted in World Cup data where total mass of forwards is correlated with success (4, 5). Amongst elite youth rugby league players, quadriceps muscle hypertrophy is related to enhancement of running speed (6). However, this may not be appropriate for all athletes with skeletal muscle hypertrophy possibly resulting in adverse adaptations, including a transition away from fast twitch glycolytic fibers and slower contraction velocity characteristics (7) if inappropriately prescribed. Thus, unless the increase in power proportionally exceeds any associated weight gain, performance is unlikely to be enhanced by skeletal muscle hypertrophy. Collectively there is support for the potential of skeletal muscle hypertrophy enhancing athletic performance, but individual athlete nuances must be considered by coaching personnel and training prescribed so as to facilitate adaptations in both muscle hypertrophy and power so that any associated increase in body mass does not negatively affect variables like speed (8).

The manipulation of dietary intake is common among individuals attempting to facilitate resistance training gains in strength and skeletal muscle hypertrophy. Aside from water (75%), skeletal muscle is made up of protein (20%), with the remainder from other materials including fat, glycogen, inorganic salts, and minerals (9). Given the protein content of skeletal muscle, it is perhaps not surprising resistance trained athletes emphasize the importance of dietary protein in their meal plans (10). This is also reflected in the scientific literature with significant attention given to protein focused nutritional interventions to facilitate resistance training induced adaptations (11), including manipulation of total daily dietary protein intake (12), protein dosage per meal (13–15), protein quality (16), and protein distribution (17). While a recent meta-analysis suggested dietary protein supplementation enhances resistance training induced gains in muscle mass and strength, at least when dietary protein intake is suboptimal (<1.6 g·kg^−1^ daily) (18), resistance training alone provides a far greater stimulus than protein supplementation (14). Given this, a number of other dietary strategies have previously been proposed to augment the resistance training response, including the creation of a positive energy balance (19, 20). While facilitating a positive energy balance is not supported by others because of the potential for increments in fat mass (FM) (21), there is clear evidence of a whole body anabolic response to overfeeding, even in the absence of a resistance training stimulus in sedentary populations (22, 23). This raises a question about composition of the lean mass accretion in this scenario i.e., skeletal muscle vs. splanchnic protein (24), especially given the lack of change in mammalian target of rapamycin (mTOR) (25). Furthermore, additional energy does not appear to further modulate the acute muscle protein synthesis (MPS) response to dietary protein ingestion at rest (26), or following resistance exercise (27, 28). Despite this, numerous textbooks used in the training of nutrition professionals advocate the creation of an energy surplus when attempting to facilitate skeletal muscle hypertrophy (29–32).

The exact energy cost of skeletal muscle hypertrophy is not known. Likewise, it is not clear if this energy cost can be met purely from endogenous (i.e., internal fat stores) and/or exogenous sources (i.e., diet). Indeed, there is clear evidence of marked skeletal muscle hypertrophy in response to a novel resistance training stimulus in otherwise healthy, overweight individuals in conjunction with a hypoenergetic, higher protein meal plan (33, 34). While similar concurrent reductions in FM and gains in FFM have been observed in elite and professional athletes following return to sport after an off-season break (35) or injury (36, 37), this response is less evident in highly trained individuals exposed regularly to a resistance training stimulus (38). This raises the possibility that individual nuances may need to be considered, including energy status and training history. Indeed, there is research confirming initial body fat stores influence metabolic response to starvation (39), while individuals with higher FFM and cardiorespiratory fitness gain less FM relative to FFM during isoenergetic, isonitrogenous overfeeding in a sedentary state (40). Preliminary research indicates younger athletes experience more pronounced physique and physical characteristic training adaptations compared to their older peers (41), a trend also even amongst mature professional athletes (38). A better appreciation of these individual nuances may assist with establishing realistic training adaptation aspirations, plus prescription of training and diet interventions to facilitate skeletal muscle hypertrophy.

It is not known if any adjustment in dietary intake to support muscle hypertrophy is required to merely contribute the building blocks of skeletal muscle while also accounting for the metabolic cost of generating new skeletal muscle mass (SMM), or if the physiological response to an energy surplus amplifies the anabolic signal created by resistance training. Addressing these fundamental questions is paramount to future prescription of energy intake guidance associated with dietary strategies to optimize skeletal muscle hypertrophy. Given the dearth of research specifically examining the influence of an energy surplus on resistance training outcomes, an exploration of overfeeding studies independent of resistance exercise has also been included in this review. This needs to be considered, given the impact of resistance training on sensitivity to nutrition support. Indeed, a single resistance training session can serve to potentiate MPS in response to protein feeding (42), an effect which may persist for upwards of 24–48 h after resistance exercise (43, 44).

Overfeeding alone is not sufficient to produce favorable body composition changes such that proportionally more FFM is gained than FM. Indeed, while 100 days of energy surplus (totaling 353 MJ) among young lean males resulted in significant individual variation in body composition change, ~2 kg of FM were accrued for each 1 kg of lean mass (45). In Leaf and Antonio's summary of overfeeding studies, they also note that predominantly more FM is gained with overfeeding in the absence of resistance training (46). However, it seems unlikely that overfeeding alone would produce meaningful increases in contractile tissue as the initiating event which induces skeletal muscle hypertrophy after maturation is the production of sufficient tension (47) and subsequent mechanotransduction at the muscle fiber level (48). In an exercise or strength and conditioning setting, this stimulus is supplied via progressive resistance training. Other related factors such as the resultant muscle damage, metabolic fatigue, and hormonal response to resistance training are speculated to either correlate with, be additive to, or play a permissive role in training-induced hypertrophy, but are not yet fully understood (49). It is plausible that nutrition could influence some of these factors.

This review not only addresses the impact of energy balance on resistance training outcomes, with an emphasis on skeletal muscle hypertrophy, but also explores other important issues, including “how big should the energy surplus be,” “where should the extra energy come from,” and “when should this extra energy be consumed.” Having a broader understanding of these issues will help establish nutrition strategies to optimize resistance training adaptations and at the same time address nutritional issues such as optimizing recovery and fuelling goals. A broader understanding of the physiological implications of an energy surplus not only has clear application to the resistance trained athlete but may also be applicable to clinical populations where retention or promotion of SMM may be advantageous. While it is recognized supplement use is common amongst resistance trained athletes (50), and there is empirical evidence to support the use of supplements like creatine monohydrate in facilitating resistance training adaptations (51), the focus of this review remains with exploring the impact of energy balance on resistance training outcomes.

## Energy Balance

The daily energy cost of protein turnover accounts for ~20% of resting energy needs or 18 kJ·kg^−1^ body mass (52). Skeletal muscle hypertrophy requires the further remodeling of muscle, ensuring it is an energy intensive process. As such, there has been much discussion around the role of energy balance (i.e., energy surpluses, energy deficits, and isocaloric states) in modulating hypertrophy. Currently, there is a paucity of literature that directly addresses the precise role energy deficits, surpluses, and net balance states play in muscle hypertrophy.

Only a few studies have directly assessed the role of energy balance on skeletal muscle hypertrophy in response to resistance training and these focus specifically on the impact of an energy deficit. Indeed, an acute, moderate energy deficit (~80% of estimated energy requirements) that promoted ~1.0 kg weight loss over 10 days amongst young healthy volunteers resulted in a 16% reduction in MPS at rest despite moderate dietary protein intake (1.5 g·kg^−1^·day^−1^), with corresponding reductions in signaling pathways involved in the protein translation protein E4-EBP1 (53). Similar findings were observed following 5 days of energy restriction (energy availability of 30 kcal·kg FFM^−1^·day^−1^), resulting in ~30% reduction in MPS amongst a group of young resistance trained volunteers, with corresponding reductions in activation of mTOR and P70S6K, protein kinases that regulate protein synthesis (54). However, a single resistance training session was able to restore MPS to levels observed in energy balance and this was further enhanced by protein ingestion (15–30 g) post-exercise, resulting in elevation of MPS ~30% above those observed at rest when in energy balance. Taken together, these acute investigations confirm an energy deficit can impair the molecular machinery involved in protein synthesis, but the overall impact on MPS will depend on other relevant factors such as dietary protein intake and resistance exercise.

The complex interaction between resistance training and diet in an energy deficit has also been explored chronically. In one study, 21 obese women were randomized to either a control arm or a resistance training arm and fed a very low energy liquid formula diet (3,369 kJ·day^−1^ containing 80 g protein, 97 g carbohydrate, 10 g fat) for 90 days. The control group and weight training group lost 16.2 and 16.8% of their body mass, respectively. Changes in body mass, FM, and FFM were similar between groups. However, muscle biopsies revealed an increase in the cross-sectional area of fast twitch muscle fibers (55). In another study on 31 women (69 ± 12 kg, 164 ± 6 cm) who engaged in 24 weeks of combined resistance and endurance training found that the cross-sectional area of thigh muscle, measured by magnetic resonance imaging, increased 7 cm, despite a 2.2% loss in body mass throughout the study (56). Similar gains in lean body mass (LBM) have been observed amongst resistance training naive overweight males in response to regular training (6 days per week, including two resistance training sessions weekly) and a higher protein diet (2.4 g·kg^−1^·day^−1^), despite a substantial energy deficit (~60% of estimated energy requirements) (34). Thus, skeletal muscle hypertrophy is possible in an energy deficit, but we propose this response may be more likely among resistance training naive, overweight, or obese individuals. The influence of training status on resistance training response to adjustments in energy balance warrants further investigation.

To our knowledge, there are no rigorously controlled investigations to date that have directly assessed the role of an energy surplus on resistance training outcomes such as skeletal muscle hypertrophy and strength/power traits over an extended period of time. However, there is an array of circumferential evidence to support the idea that an energy surplus does enhance gains in FFM, even independent of the resistance training stimulus. In an early overfeeding study in which 12 pairs of identical male twins were fed a total energy surplus of 353 MJ (with 15% of total daily energy intake from protein) over a span of 100 days (or 4,200 kJ·day^−1^), the volunteers gained an average of 5.4 kg FM and 2.7 kg FFM (45), despite maintaining a relatively sedentary lifestyle. There was a high level of intra-pair correlation among twins, but significant variance between groups of twins, indicating a significant genetic contribution to the adaptation. Another overfeeding study explored the effect of a similar energy surplus (~40% above estimated daily needs or ~4,000 kJ energy surplus daily) but with varying levels of protein intake (5, 15, or 25% of total energy intake) on body composition over an 8-week period. While all groups increased body fat by similar amounts (~3.5 kg) during this tightly controlled metabolic unit investigation, gains in LBM (~3 kg) were only evident with the two higher protein intakes, suggesting a minimum amount of dietary protein is necessary to facilitate gains in LBM, even in an energy surplus (23).

In a preliminary exploration of the combined effects of an energy surplus and resistance training, it was found that only those individuals who consumed an energy dense liquid supplement twice daily on training days observed significant gains in body mass and FFM, as inferred via hydrodensitometry, over an 8-week training period (57). Furthermore, there was no difference in response whether the extra energy was consumed as carbohydrate or a combination of carbohydrate and protein, suggesting the energy content of the diet had the biggest impact on body composition changes when dietary protein intake is already adequate. This is supported by earlier pilot work on the influence of an energy surplus on resistance training adaptations (58). Interestingly, while both investigations confirmed a favorable influence of an energy surplus on FFM gains, this was not reflected in strength changes, perhaps because of the brief duration of training or due to nuances in the techniques used to assess strength and body composition. A recently published pilot study on male bodybuilders also supports the concept of greater body mass and muscle mass gains with a more aggressive energy intake (282 kJ·kg^−1^·day^−1^), although further inferences from this study are difficult due to methodological concerns (59).

While much still needs to be done to understand the precise role an energy surplus has in facilitating skeletal muscle hypertrophy, the following discussion explores what is known about the magnitude of a surplus, macronutrient composition, and the mechanisms surrounding the role an energy surplus has on skeletal muscle hypertrophy. Given the lack of research within this environment, exploration of overfeeding studies, independent of the resistance training stimulus, are included in the discussion on this topic. This needs to be considered given the influence resistance training has on protein metabolism, highlighting the symbiotic influence of training, and diet on resistance training adaptations.

## Energy Surplus… How Much

Common text book recommendations for the energy surplus required to gain 1 kg of SMM range from ~1,500 to 2,000 kJ·day^−1^ in weight stable athletes to an additional 4,000 kJ·day^−1^ in individuals who struggle with lean mass gains or during heavy training loads (31, 32). Guidance on the energy surplus necessary to facilitate skeletal muscle hypertrophy is often based solely on the foundation that if 1 kg of skeletal muscle is 75% water, 20% protein, and 5% fat, glycogen and other minerals and metabolites, then the energy required to accumulate such tissue must at a minimum equal the sum of its parts. Given the assumed composition of skeletal muscle, the energy stored in 1 kg of muscle is ~5,000–5,200 kJ, with ~3,400 kJ from protein, ~1,400–1,500 kJ from fat, and ~300–450 kJ from muscle glycogen. Furthermore, energy intake should also be sufficient to supply substrate to fuel the protein synthetic machinery stimulated by resistance exercise, a potentially costly process (11, 60). Finally, adequate energy may be needed to account for the increased metabolic cost of accumulated muscle mass and diet induced thermogenesis (DIT), all while minimizing additional energy stored as FM. However, the foundations of these estimates fail to recognize the complicated and energetically expensive process of tissue accretion, an energy value which remains to be systematically quantified. To date, the authors are not aware of any studies that have clearly demonstrated a consistent energy cost of tissue accumulation, specifically that which is associated with skeletal muscle hypertrophy in response to a resistance training stimulus.

Throughout the twentieth century numerous obesity researchers investigated the influence an energy surplus has on body composition. Most studies consistently demonstrate a strong association between body mass gain and the energy surplus (61). However, there is large inter-individual variability in the composition of this mass gain with between 33 and 40% of body mass accretion accounted for by increases in FFM (61). In non-exercising populations some have suggested that the composition of tissue change associated with an energy surplus is a fixed relationship (62), but in athletic populations where exercise and adequate protein intake are the main stimuli for SMM adaptation, this seems unlikely. Of interest from this obesity research is Forbes and colleagues attempt to estimate the energy cost of tissue deposition by using theoretical values of deposition and comparing them to their own findings (22). They reported that by using the values suggested by Spady et al. (63), which were 36.2 kJ·g^−1^ of protein and 50.2 kJ·g^−1^ of fat deposited, in combination with composition ratios of FM:FFM observed in their research, that the energy cost of depositing 1 kg was closely aligned with theoretical values (31,600 and 33,800 kJ·kg^−1^, respectively). As part of this, Forbes surmised that due to SMM being 20% protein and 75% water, the energy cost of depositing 1 kg of SMM was 7,440 kJ·kg^−1^. More recently, Joosen and Westerp (61) have suggested a figure of 29.4 kJ·g^−1^ of protein deposition, potentially reducing the cost of SMM deposition to 6,050 kJ·kg^−1^. Both estimates suggest an additional energy cost to deposit tissue above the energy density of the substrate (i.e., 16.7 kJ·g^−1^ for protein) of between 12.7 and 19.5 kJ·g^−1^ protein after overfeeding in non-exercising individuals.

As previously highlighted, research suggests considerable inter-individual variability in body mass and composition changes associated with an energy surplus, perhaps as a consequence of genetics (40, 45) or metabolic responses such as adaptation to DIT or non-exercise activity thermogenesis (NEAT) (18, 64). Numerous mathematical models have been published to predict changes in body composition associated with changes in dietary intake and energy expenditure (65, 66). However, these equations often standardize estimates of whole-body metabolic energy flux due to the non-exercising population they are focused on (e.g., no change in glycogen state over time, constant relationships between FM and FFM based on population norms). In athletic populations, the nature of training for body composition alterations significantly influences exercise energy expenditure and confounds the ratios of tissue deposition these models rely on. Therefore, if practitioners are to provide guidelines on the energy surplus necessary to synthesize 1 kg of SMM with minimal FM change, it is necessary that a more expansive model of energy cost be explored.

**Figure 1:**
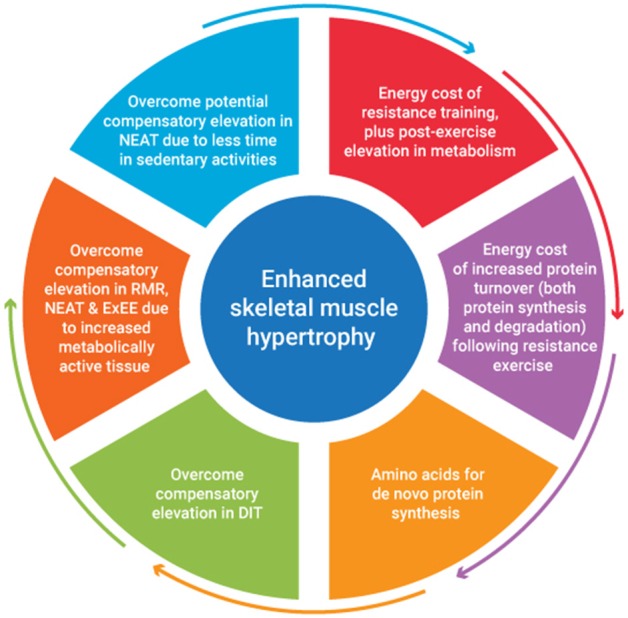
An overview of factors contributing to the energy cost of skeletal muscle hypertrophy.

A recent review of studies investigating the combination of a protein focused energy surplus with resistance training have indicated favorable improvements in LBM accretion (46). However, to date few studies have focused on a titrated energy surplus to ascertain the exact energy and nutrient cost of SMM accretion. It seems to the authors that the energy cost of SMM accretion would be accounted for by consideration of several issues. These include the energy stored within muscle tissue, the energy cost of resistance exercise plus any associated post-exercise elevation in metabolism, the energy cost of any subsequent tissue generation, plus it's subsequent metabolic function. The metabolic adjustments that occur in response to an energy surplus also need to be considered. Figure 1 provides an overview of factors contributing to the energy cost of skeletal muscle hypertrophy. An appreciation of the magnitude of these factors would provide greater insight into appropriate energy intake prescription to facilitate quality weight gain i.e., weight gain characterized primarily by gains in FFM.

Numerous studies have attempted to estimate the energy cost of single (67), multiple set (68), and varying speed and intensity (69) resistance exercise sessions, with the net energy cost of an 8 exercise (2 sets of 8–10 repetitions per exercise) hypertrophy program lasting ~30 min being ~300 and 600 kJ, for females and males, respectively (70, 71). These sex differences in net energy expenditure are not evident when normalized for lean mass (72). Given the potential importance of quantifying energy expenditure, estimates of net resistance training energy expenditure are available i.e., total energy expenditure (TEE) minus resting energy expenditure or the specific energy cost of the resistance training alone. Mookerjee et al. (68) report the energy cost of undertaking 3 sets of 10 reps at 70% of one repetition maximum across five upper body exercises (369.4 ± 174.1 kJ), equating to an energy expenditure of ~0.10–0.12 kJ·kg^−1^ LBM·min^−1^. More recently, a regression equation has been established to estimate resistance training relative energy expenditure based on several variables including stature, age, FM, LBM, and total exercise volume (72), giving practitioners several options to assist with quantifying the energy cost of resistance training. While some of these estimates fail to account for any elevation in energy expenditure post exercise, this effect may only be evident for upwards of 20 min post exercise (72), and thus may be considered negligible, at least following shorter duration resistance training. The metabolic implications of resistance training warrant further exploration.

Given skeletal MPS is elevated for upwards of 24–48 h after resistance exercise, the high metabolic cost of protein synthesis needs to also be accounted for (60, 73, 74). The process of translation elongation is likely to account for a large portion of the synthetic cost with 4 high energy phosphate bonds per peptide bond formed required or 3.6 kJ·g^−1^ of protein synthesized (75). Although significant, translation is one of many energy requiring steps in protein synthesis, with processes such as transcription, folding and movement of proteins within cells all being energy dependent (52). The high energy cost of protein synthesis and the duration over which protein synthetic machinery can be upregulated clearly highlights an underestimated cost of protein synthesis and thus, muscle mass accretion. While any associated increase in protein breakdown has been considered to be negligible, this is unlikely the case (52). Further research is needed to better quantify the energy cost of protein synthesis and degradation, plus the time frame over which this may impact energy needs.

Any increase in LBM will also increase energy expenditure, both at rest and during exercise due to the addition of metabolically active tissue, but the implications of this are likely substantially less than is often presumed. Indeed, estimates of the metabolic activity of individual components of FFM suggest skeletal muscle has an energy cost of just 54 kJ·kg^−1^·day^−1^ (76, 77). Given this, the elevation in REE in response to a 1–2 kg gain in muscle mass is likely very small (i.e., ~100 kJ), and within the precision error of indirect calorimetry techniques available to quantify REE (78). Less is known about the impact of both training and an energy surplus on high metabolic activity tissues like internal organs. However, just 3 weeks of energy restriction has been shown to significantly decrease liver and kidney mass, with associated reductions in REE (79), confirming manipulation of energy balance may impact high metabolic activity tissue size and thus presumably energy expenditure.

Any adjustment in energy intake away from energy balance results in an adaptive change in energy expenditure, via adjustments in NEAT, DIT, and/or adaptive thermogenesis (AT). Indeed, energy expenditure has been observed to increase after just 24 h in an energy surplus (40% above estimated needs), at least when protein intake is concomitantly increased (25). It has also been proposed that overfeeding induces an increase in heat production from the food consumed as a protective mechanism against obesity, a process termed luxuskonsumption which is claimed to dissipate upwards of 30% of the excess energy consumed (64). This form of AT has more recently been challenged by Muller et al. (80) with their assessment of overfeeding literature suggesting in most studies 60–70% of excess energy was stored and a further 20–30% accounted for by metabolic lean mass accretion and increased cost of movement leaving only about 10% of energy expenditure not explained and likely accounted for by errors of measurement. One component of TEE that increases based on changes to the baseline food consumption is DIT. The DIT associated with a typical western diet accounts for ~8–15% of TEE, depending on the macronutrient breakdown of the diet (81). In a review of 26 studies investigating DIT, Quatela et al. (82) used a mixed model meta-regression process to estimate DIT associated with overfeeding. They suggested for every 100 kJ of additional energy from a mixed diet, DIT increased by 1.1 kJ·h^−1^. Energy surpluses associated with higher protein intakes >3.0 g·kg^−1^ BM are likely to increase this figure further and potentially add an additional energy requirement compared to surpluses with energy coming from carbohydrate and fat. This could require an additional 500 kJ a day for athletes with protein intakes in the rage of 3.0 g·kg^−1^·day^−1^ compared to intakes of 1.0 g·kg^−1^·day^−1^. Another significant component of TEE that is highly variable in exercising individuals, and may be influenced by training and eating, is NEAT (83). Levine et al. (84) observed a significant increase in NEAT (1,380 ± 1,080 kJ·day^−1^) among individuals over feed 4,200 kJ·day^−1^ for 56 days. Although there are some arguments against this response (64), it is likely this component of TEE may be highly variable among individuals with different physical activity levels when in an energy surplus.

Finally, the influence dietary energy intake has on the anabolic hormonal environment is becoming better understood. It is now well-established that energy restriction can significantly influence anabolic hormones in exercising individuals, potentially impairing their ability to gain and maintain LBM (85). Although early research by Forbes and colleagues suggested that an energy surplus could improve anabolic hormone levels in women (86), few other studies have demonstrated significant increases in the hormonal environment in response to an energy surplus (87, 88). Irrespective, such acute elevations in circulating anabolic hormones may have little, if any, impact on resistance training adaptations (89, 90). Thus, any benefit of an energy surplus is likely mediated via mechanisms other than acutely influencing the anabolic hormonal environment.

**Figure 2:**
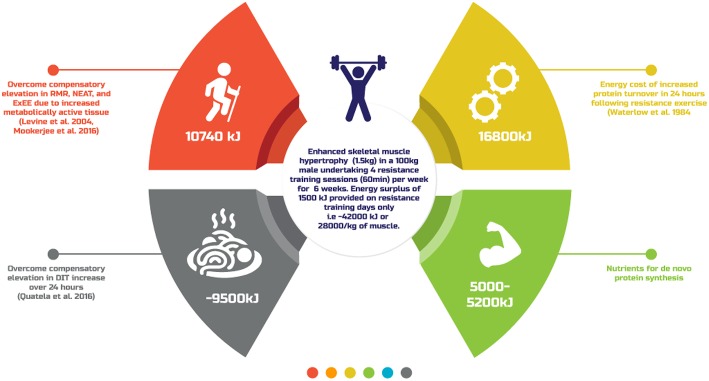
A theoretical overview of the energy cost of generating skeletal muscle mass typically reported amongst resistance training individuals.

What is clear from the existing literature is that there is yet to be defined a single evidence-based energy estimate for accretion of 1 kg of SMM. This is most likely because of the impact of individual presenting nuances (age, genetics, prior training experience, sex, body composition) as well as adaptation to the energy surplus. Figure 2 provides a theoretical overview of the energy cost of generating SMM typically reported amongst resistance training individuals (91), the results of which are similar to that estimated previously (22). A better understanding of these variables and their impact on resistance training induced skeletal muscle hypertrophy may afford better individual prescription of the energy surplus. Until then, practitioners are advised to take a conservative approach to creating an energy surplus, within the range of ~1,500–2,000 kJ·day^−1^, to minimize FM gains, with regular review of body composition and functional capacities like strength to further personalize dietary intake.

## Energy Surplus… Macronutrient Considerations

Within the constraints of an athlete's total daily energy intake, considering appropriate allocation of protein, carbohydrate, and fat may also have a measurable impact on skeletal muscle hypertrophy. Dietary protein has long been identified as a critical macronutrient to consider in skeletal muscle repair and synthesis. Indeed, resistance trained athletes have advocated high protein diets for many years (10). While debate continues on the need for additional protein amongst athletes, general guidelines now recommend that athletes undertaking resistance training ingest approximately twice the current recommendations for protein of their sedentary counterparts or as much as 1.6–2.2 g·kg^−1^·day^−1^ (92). In a recent meta-regression of 49 studies including 1,863 male and female participants, the protein intake associated with the greatest gains in muscle mass was 1.6 g·kg^−1^·day^−1^ (18). Exceeding this upper range of protein intake guidelines likely offers no further benefit and simply promotes increased amino acid catabolism and protein oxidation (14). Even extremely high protein intakes, up to double that advocated (18), does not further facilitate skeletal muscle hypertrophy or strength gains (93–95).

Despite a lack of apparent benefits from a high protein diet, athletes who are particularly sensitive to gains in FM may be tempted to source additional energy to facilitate an energy surplus from protein, given it is suggested to be less lipogenic, presumably because of increased DIT (96). However, the impact on DIT is small in absolute terms and thus unlikely to significantly influence the response to an energy surplus (97). While it has been claimed protein, and thus energy intake, can be increased substantially without promoting gains in FM (93–95, 98), it is difficult to understand how this is possible, even after considering the impact on DIT. Indeed, tightly controlled research exploring the impact of variable protein intakes while overfeeding in a metabolic hospital ward confirms protein intake impacts lean mass while the energy surplus alone contributes to increases in FM, at least amongst sedentary individuals (23).

Despite previous concerns that high protein diets may be harmful, healthy adults with protein intakes of 1.8 g·kg^−1^·day^−1^ show no adverse effect on renal function (99). Furthermore, very high protein diets (2.5–3.3 g·kg^−1^·day^−1^) consumed over a year had no deleterious effects on blood lipids, liver, or renal function (100). However, the health implications of very high protein diets over longer periods is yet to be elucidated. Taken collectively, it is hard to justify the very high protein intakes consumed by some resistance training athletes, given the current lack of supporting research in enhancing resistance training adaptations, nor research confirming such high intakes are without health implications.

In addition to protein requirements, consideration must also be given to appropriate allocation of carbohydrate and fat in a meal plan attempting to facilitate muscle hypertrophy. Short term overfeeding studies in sedentary populations confirm there is no significant difference in body composition changes whether the energy surplus comes predominantly from carbohydrate or fat (101, 102). However, the metabolic implications of exercise must be considered amongst resistance trained individuals. Given the primary substrate used during resistance training is carbohydrate (103), it is logical to explore the provision of additional carbohydrate to help support training demands. This may be especially so for athletes other than weightlifters, powerlifters, and bodybuilders, where resistance training is typically undertaken as an ancillary form of training to complement sport specific training. Resistance training can reduce muscle glycogen stores by 30–40% (104). Therefore, larger volume, hypertrophy focused resistance training may necessitate additional carbohydrate to facilitate resistance training work capacity (105, 106) and restore muscle glycogen (107). While it is difficult to confirm further enhancement in acute training capacity (108) or chronic body composition adaptations (58), when contrasting a moderate vs. high carbohydrate intake, a chronic restrictive carbohydrate intake may impair resistance training adaptations. Indeed, SMM gains have consistently been impaired in studies of resistance trained individuals following high fat, “ketogenic” diets when compared to moderate intakes (109–111). Given this, it seems reasonable to continue to support carbohydrate intakes within the range of 4–7 g·kg^−1^·day^−1^ for strength trained athletes (112), with upper ranges advocated for those undertaking resistance exercise as an ancillary form of training to complement sport specific training.

The American College of Sports Medicine advises athletes to keep fat intakes in line with general health guidelines (113), which constitutes 20–35% of energy intake. Athletes should be discouraged from fat intakes below 15–20% of energy intake since such restrictions likely moderate the energy density of a meal plan, making it challenging to facilitate an energy surplus while also reducing intake and absorption of fat-soluble vitamins (114, 115). Furthermore, reducing dietary fat from 33.3 to 13.9% of total energy intake resulted in modest but significant reductions in resting testosterone concentrations (116), a result which has been replicated elsewhere (117, 118). However, the relevance of these small changes in circulating androgens is unknown in the context of chronic resistance training adaptation.

Given the energy density of fat is effectively double that of carbohydrate and protein, it is logical to consider increasing fat intake when attempting to increase the energy density of a meal plan. Indeed, within hypermetabolic clinical conditions such as cystic fibrosis requiring a high energy intake, increasing fat intake is advocated (119). Fat source may also determine the fate of excess energy, with polyunsaturated fat more likely to promote gains in lean mass compared to saturated fat, which is more likely to result in ectopic and general fat accumulation in normal weight volunteers (120). Evolving evidence suggests omega-3 polyunsaturated fatty acid ingestion enhances the anabolic response to nutritional stimuli and increases muscle mass and function in young and middle-aged males (121), plus older adults (122), respectively, independent of the resistance training stimuli. There are also health benefits to consider in the type of fat consumed. Postprandial fat oxidation is higher after monounsaturated (olive oil) compared to saturated (cream) fat meals (123). Simply substituting saturated fat for unsaturated fat, predominantly as monounsaturated fat, was enough to induce favorable improvements in lipid profile and reductions in fat mass in a small sample of overweight and obese males (124). Whilst interesting, further research is required to determine whether the potential benefits in the type of fat ingested are maintained in an athletic population undergoing resistance training in an energy surplus and whether this influences the quality of weight gain. Until then, international recommendations indicate that active individuals may consume up to 35% of their daily energy intake from dietary fat, with saturated fatty acids not exceeding 10% of total energy intake (114).

The type of foods from which macronutrients are sourced may also have implications on lean mass gains. Protein type is important as high biological value protein sources rich in leucine are recommended to maximize protein synthetic rates (125). Consumption of protein in its natural whole-food matrix may also differentially stimulate muscle anabolic properties compared to isolated proteins particularly post resistance training (126). This has been observed with whole milk compared to skim milk (127), and whole eggs compared to egg whites (126). Thus, additional nutrients found in whole foods may offer advantages beyond their amino acid profile to maximize protein synthesis (128), although more research is required to ascertain how this occurs and if benefits remain when total dietary protein and energy is matched.

In conclusion, insufficient data exists to promote an energy surplus that comes primarily from any specific macronutrient. Thus, without further research we can only emphasize that the minimum intakes of macronutrients advised in this section be achieved while ensuring an appropriate energy surplus. Preliminary evidence suggests extra protein may be less lipogenic, perhaps because of an increase in energy expenditure associated with DIT, although this needs to be confirmed with better controlled studies on resistance training populations and may need to merely be corrected by further increasing energy intake if the same energy surplus is desired. Furthermore, the health implications of sustained protein intakes above ~2.5 g·kg^−1^·day^−1^ remain to be validated. As such, other factors such as individual preference, allocation of extra energy over the day relative to resistance training, existing energy density of the meal plan and potential for increasing the volume of existing food/ fluid intake may be a higher priority when considering the source of any prescribed energy surplus.

## Energy Surplus… Nutrient Timing

Nutrient timing has received significant attention in recent years (129), with interventions aiming to optimize work capacity during exercise and/or facilitate training adaptations. Specifically, primary attention has been given to the timing of protein and carbohydrate intake to support acute fuelling and recovery goals (130), plus facilitate chronic skeletal muscle hypertrophy adaptations (18). However, whenever daily macronutrient distribution is adjusted, so too potentially is energy intake. Thus, the influence of daily energy distribution, including the number of eating occasions, also warrants consideration.

Athletes are encouraged to pay attention to dietary intake pre, during and post exercise, under the assumption that nutritional strategies can influence both acute resistance exercise capacity and/ or training induced adaptations. Indeed, evidence is present for a beneficial role of acute carbohydrate ingestion before and/ or during strength training (105, 131). However, not all investigations show a benefit of acute carbohydrate ingestion (132–134), suggesting the ergogenic potential for carbohydrate ingestion is most likely to be observed when athletes are undertaking longer-duration, high-volume resistance training in isolation, or when resistance training is incorporated into a higher volume total training load that also includes sports specific training. Currently, specific recommendations for an optimum rate or timing of carbohydrate ingestion for resistance trained athletes before and during a resistance training session cannot be made within broader guidance of 4–7 g·kg^−1^ body mass daily (112). However, this warrants investigation given the potential for enhanced substrate availability, plus better alignment of energy intake to expenditure.

The consumption of high biological value protein containing meals/snacks in close proximity to training is widely applied as a strategy to augment the skeletal muscle adaptive response to resistance exercise (135). Less is known about the impact of protein distribution in the meal plan outside of the acute period before and/or after exercise (<3 h). There is some evidence to suggest that skeletal MPS may be enhanced with a wider distribution of daily protein intake compared with an acute bolus of protein (17). Indeed, spacing protein-containing meals (~0.3 g·kg^−1^ of high biological value protein) every 3–5 h throughout the waking period of the day has been advocated when attempting to maximizes MPS (92), although this remains to be validated amongst resistance trained individuals when ingesting protein as part of mixed macronutrient meals while in energy balance or surplus. Indeed, increasing daily distribution of high biological value protein from four to six meals per day had no influence of pre-season gains in lean body mass amongst a group of rugby athletes (91), suggesting a threshold of daily protein containing meals, above which there is likely no further enhancement in skeletal muscle hypertrophy when in energy balance/surplus, perhaps due to the hypothesized refractory period that follows acute protein ingestion (136).

While skeletal MPS is unlikely to be further enhanced by more frequent eating occasions, smaller more frequent eating occasions (5–6+) are advocated when attempting to increase muscle mass, presumably because gastrointestinal tract tolerance is higher with more frequent eating occasions compared to merely increasing the size of existing eating occasions (31). Indeed, smaller, more frequent meals are advocated clinically in the management of early satiety, anorexia and gastrointestinal symptoms (137). Emerging evidence supports this notion, with significantly stronger hunger and desire to eat when following a smaller, more frequent eating pattern (138). This is corroborated by preliminary data in elite athletes, with a moderate association between meal frequency and total energy intake (139). Given that snacks accounted for approximately one-quarter of total energy intake in this athletic population, it seems pertinent to advocate the inclusion of snacks in the meal pattern of athletes attempting to increase overall energy intake. Current evidence suggests athletes ingest food daily typically over ~5 eating occasions, including the three main meals, plus snacks (139, 140). The impact of eating occasion frequency on overall nutrient intake and subsequent resistance training outcomes warrants investigation in athletic populations. Until then, athletes are encouraged to consume a minimum of 3 main meals, with the use of strategic snacks to support fuelling and recovery goals, plus facilitate skeletal MPS.

Similar to the general population (141), athletes allocate more of their daily energy intake to the later part of the day (140, 142). The impact of better alignment of daily energy intake to expenditure, or within day energy balance, is an emerging area of research interest focused on the physiological implications of real-time changes in energy intake and expenditure. Preliminary research suggests unfavorable metabolic and endocrine perturbations with large acute or extended energy deficits amongst athletes focused on leanness (143–145). The implications of manipulating within day energy balance amongst resistance training athletes attempting to promote quality weight gain has not been investigated but warrants consideration.

There is some preliminary evidence to suggest better alignment of energy intake to expenditure may have application in facilitating resistance training outcomes. Ingestion of a creatine monohydrate containing carbohydrate-protein supplement immediately before and after resistance training results in more favorable resistance training adaptations than when the same supplement is ingested away from training (146), although this is not always evident, at least when a lower energy, protein only supplement is ingested according to a similar time frame (147). While it is impossible to ascribe this effect to the timing of macronutrient or energy intake, this approach toward optimizing nutrition support before and after a resistance training session also supports general fuelling and recovery goals. It also better aligns acute energy intake to expenditure, given daily energy expenditure is likely highest during exercise.

For athletes focused on facilitating quality weight gain, consideration of temporal energy patterns may also be warranted given preliminary research suggesting an association between eating more of the day's total energy intake at night and obesity (148, 149). This may be due to the metabolic dysfunction induced by delayed eating, even amongst normal weight individuals (150), or it may merely reflect behavioral mechanisms that influence appetite control (151). Indeed, intake in the late night also appears to lack satiating value, resulting in greater overall daily intake (152). While tempting to advocate athletes to “front end” more of the daily energy intake, especially amongst individuals aiming to minimize fat mass gains, moderating energy intake as the day progresses may be inappropriate for those with high energy needs and/ or those with significant training commitments in the evening. Indeed, there is evidence of enhanced strength and muscle mass gains from resistance training undertaken in the evening when two protein containing meals are ingested prior to bed compared to one (153). As such, manipulation of daily energy distribution should merely be a variable practitioners consider when providing advice to athletes, adjusting according to the individual athlete and their unique circumstances, including specific energy needs, timing of training, and nutrition goals. The influence of daily energy distribution warrants investigation amongst resistance trained athletes attempting to facilitate quality weight gain.

While it is logical to encourage energy intake to vary over a training week to reflect exercise energy expenditure, athletes do not always adjust intake to reflect expenditure (154), perhaps in part because of the variable impact exercise has on appetite (155). If the creation of a positive energy balance is desired to facilitate resistance training adaptations, one variable to consider is whether that energy surplus should be applied throughout the week or just on resistance training days. Supplemental energy has typically only been provided on resistance training days in the limited research in which a positive energy balance has been achieved in conjunction with resistance training (57). While this better mirrors energy intake to expenditure, it could be argued given that skeletal MPS is elevated for upwards of 48 h following a single resistance training session (43), that a positive energy balance is also warranted for upwards of 24–48 h post training. Presumably the creation of a positive energy balance on both resistance training and non-training days may help to optimize the potential for enhanced skeletal muscle hypertrophy. This issue warrants further investigation in resistance trained populations, especially amongst those individuals aiming to facilitate quality weight gain. The concept of intermittent energy restriction shows preliminary potential for facilitating more effective quality weight loss by moderating any associated metabolic adjustments (156). It would be interesting to explore if the reverse was also true with the intermittent application of a positive energy balance for facilitating quality weight gain. Indeed, there is preliminary evidence to suggest an acute energy surplus (facilitated via an increase in all macronutrients) results in preferential gains in fat free mass (157).

## Managing Satiety

Attempts to increase total energy intake by merely increasing the total volume of food ingested may result in early satiety, limiting the potential for creation of an energy surplus. Thus, consideration may need to be given to increasing the energy density of the meal plan. While increasing dietary fat intake is a logical option, other novel strategies to better manage early satiety include changing the food form. For example, regardless of the predominant energy source, drinks have lower satiety than solid foods and thus, provide greater potential for facilitating a positive energy balance (158, 159). Furthermore, nutritious drinks can be particularly practical following exercise when the appetite may be suppressed, while also supporting nutritional recovery goals. A high intake of low energy density vegetables may also moderate total energy intake at a meal (160). However, given the health benefits of individuals achieving public health guidance on vegetable intake (161), practitioners are advised to balance the pursuit of enhancing energy density with overall health benefits of the meal plan. Advocating the ingestion of only moderate servings of protein rich foods at meals may also be appropriate, given the satiating effect of protein (162), although the implications of higher protein meals on satiety when in a positive energy balance remain to be confirmed. However, given moderate protein servings will also help optimize the skeletal muscle protein synthetic response (92), guidance on moderated protein servings appears logical.

## Future Directions

Further research into this area is clearly warranted, but challenged by individual responsiveness, including the potential for rapid metabolic adjustment to the energy surplus and the need to consider not only the energy surplus, but potentially where that energy comes from and how it is allocated in the meal plan over the day relative to the resistance training stimulus. Methodological issues associated with the quantification of key outcome measures such as energy intake, energy expenditure and body composition are also very relevant when attempting to interpret the literature. For example, an increase in dietary carbohydrate intake to facilitate a positive energy balance will acutely increase muscle metabolites and associated water content, significantly influencing estimates of body composition via dual energy x-ray absorptiometry (163), and other commonly used techniques, including air displacement plethysmography and bioimpedance analysis (164), while acute resistance exercise induced water retention can influence magnetic resonance imaging estimates of muscle cross-sectional area for at least 52 h (165). Given such physique assessment nuances, concurrent review of associated functional capacity adaptations would appear pertinent for future investigations.

Several gaps in the literature have been identified in this review, which warrant further exploration. Some of these are expanded upon here in the hope of facilitating research interest in this area. A broader understanding of these issues has the potential to not only impact on dietary guidance for athletic populations, but also clinical populations where retention or promotion of SMM is advocated.

### Should the Prescribed Energy Surplus Be Adjusted Based on the Anticipated Muscle Hypertrophy Potential of the Athlete?

Younger, less experienced athletes have a greater potential for skeletal muscle hypertrophy in response to resistance training than their more experienced counterparts (38). It could be argued that if the energy surplus is merely required to contribute the building blocks of newly generated tissue, then the prescribed energy surplus should be adjusted based on muscle hypertrophy potential. Preliminary research in a small group of elite Norwegian athletes supports this hypothesis (166). However, if the energy surplus facilitates a physiological response that amplifies the anabolic signal created by resistance training, then perhaps the energy surplus should be maintained in experienced athletes, at least in these where muscle hypertrophy and strength gains are prioritized over short term FM increments.

### What Factors Influence Whether Endogenous and/or Exogenous Energy Sources Can Support the Energy Cost of Muscle Hypertrophy?

The presence of muscle hypertrophy in response to resistance training while in an energy deficit clearly confirms the energy cost of hypertrophy can be obtained endogenously (34, 55, 56), but is more likely evident amongst resistance training naïve, overweight individuals. Thus, individual nuances such as presenting energy status and training history may need to be considered when prescribing energy intake.

### Does Better Temporal Alignment of Daily Energy Intake to Expenditure (Within Day and Between Day) Result in More Favorable Gains in FFM Relative to FM When in an Energy Surplus?

While preliminary research suggests unfavorable metabolic and endocrine perturbations with large acute, within day energy deficits amongst athletes (144, 145), less is known about the potential benefit of better aligning daily energy intake to expenditure when in an energy surplus. Encouraging preliminary research indicates a more favorable response to resistance training when more of the daily energy intake is allocated immediately before and after exercise (146). The influence of better aligning daily energy intake to expenditure across a training week also warrants investigation, the results of which would help to identify if any energy surplus should be applied on raining days only or throughout the training week.

## Conclusions

The creation of an energy surplus is commonly advocated by sports nutrition practitioners when attempting to optimize resistance training induced skeletal muscle hypertrophy. Such guidance is often based solely on the assumed energy stored within the tissue being assimilated. Unfortunately, this fails to account for other energetically expensive processes, including the energy cost of tissue generation, plus the metabolic adjustments that occur in response to an energy surplus. An appreciation of the magnitude of these factors would provide greater insight into appropriate energy prescription to facilitate optimal rates of muscle hypertrophy while minimizing fat mass gain. Until that time, practitioners are advised to start conservatively with an energy surplus within the range of ~1,500–2,000 kJ·day^−1^ and closely monitor response to the intervention, using changes in body composition and functional capacity to further personalize dietary interventions. So long as minimum guidelines for macronutrients advocated for resistance training individuals are achieved, there does not appear to be any metabolic or functional benefit to the source of the energy surplus, affording the practitioner an opportunity to adjust intake based on other variables such as existing energy density of the meal plan, eating occasions and distribution of energy, and macronutrient intake relative to training, plus potential for further increasing food intake.

## Author Contributions

BD, DM, EH, GS, GJS, and JI drafted, critically reviewed, and revised the manuscript for important intellectual content, contributed to manuscript revision, read, and approved the submitted version.

### Conflict of Interest Statement

The authors declare that the research was conducted in the absence of any commercial or financial relationships that could be construed as a potential conflict of interest.

## References

[1] Brechue WF Abe T. The role of FFM accumulation and skeletal muscle architecture in powerlifting performance. Eur J Appl Physiol. (2002) 86:327–36. 10.1007/s00421-001-0543-7 11990746

[2] Siahkouhian M Hedayatneja M Correlations of anthropometric and body composition variables with the performance of young elite weightlifters. J Hum Kinet. (2010) 25:125–31. 10.2478/v10078-010-0040-3

[3] Bell W Evans WD Cobner DM Eston RG. Regional placement of bone mineral mass, fat mass, and lean soft tissue mass in young adult rugby union players. Ergonomics. (2005) 48:1462–72. 10.1080/00140130500101007 16338713

[4] Olds T. The evolution of physique in male rugby union players in the twentieth century. J Sports Sci. (2001) 19:253–62. 10.1080/026404101750158312 11311023

[5] Barr MJ Newton RU Sheppard JM Were height and mass related to performance at the 2007 and 2011 rugby world cups? Int J Sports Sci Coach. (2014) 9:671–80. 10.1260/1747-9541.9.4.671

[6] Waldron M Worsfold P Twist C Lamb K. Changes in anthropometry and performance, and their interrelationships, across three seasons in elite youth rugby league players. J Strength Cond Res. (2014) 28:3128–36. 10.1519/JSC.0000000000000445 25226320

[7] Alway SE MacDougall JD Sale DG Sutton JR McComas AJ. Functional and structural adaptations in skeletal muscle of trained athletes. J Appl Physiol. (1988) 64:1114–20. 10.1152/jappl.1988.64.3.1114 3366734

[8] Barr MJ Sheppard JM Gabbett TJ Newton RU. Long-term training-induced changes in sprinting speed and sprint momentum in elite rugby union players. J Strength Cond Res. (2014) 28:2724–31. 10.1519/JSC.0000000000000364 24402451

[9] Frontera WR Ochala J. Skeletal muscle: a brief review of structure and function. Calcif Tissue Int. (2015) 96:183–95. 10.1007/s00223-014-9915-y 25294644

[10] Mitchell L Hackett D Gifford J Estermann F O'Connor H. Do bodybuilders use evidence-based nutrition strategies to manipulate physique? Sports. (2017) 5:E76. 10.3390/sports5040076 29910436 PMC5969027

[11] Morton RW McGlory C Phillips SM. Nutritional interventions to augment resistance training-induced skeletal muscle hypertrophy. Front Physiol. (2015) 6:245. 10.3389/fphys.2015.00245 26388782 PMC4558471

[12] Tarnopolsky MA Atkinson SA MacDougall JD Chesley A Phillips S Schwarcz HP. Evaluation of protein requirements for trained strength athletes. J Appl Physiol. (1992) 73:1986–95. 10.1152/jappl.1992.73.5.1986 1474076

[13] Moore DR Robinson MJ Fry JL Tang JE Glover EI Wilkinson SB. Ingested protein dose response of muscle and albumin protein synthesis after resistance exercise in young men. Am J Clin Nutr. (2009) 89:161–8. 10.3945/ajcn.2008.26401 19056590

[14] Witard OC Jackman SR Breen L Smith K Selby A Tipton KD. Myofibrillar muscle protein synthesis rates subsequent to a meal in response to increasing doses of whey protein at rest and after resistance exercise. Am J Clin Nutr. (2014) 99:86–95. 10.3945/ajcn.112.055517 24257722

[15] Macnaughton LS Wardle SL Witard OC McGlory C Hamilton DL Jeromson S. The response of muscle protein synthesis following whole-body resistance exercise is greater following 40 g than 20 g of ingested whey protein. Physiol Rep. (2016) 4:e12893. 10.14814/phy2.12893 27511985 PMC4985555

[16] Devries MC Phillips SM. Supplemental protein in support of muscle mass and health: advantage whey. J Food Sci. (2015) 80(Suppl. 1):A8–15. 10.1111/1750-3841.12802 25757896

[17] Areta JL Burke LM Ross ML Camera DM West DW Broad EM. Timing and distribution of protein ingestion during prolonged recovery from resistance exercise alters myofibrillar protein synthesis. J Physiol. (2013) 591:2319–31. 10.1113/jphysiol.2012.244897 23459753 PMC3650697

[18] Morton RW Murphy KT McKellar SR Schoenfeld BJ Henselmans M Helms E. A systematic review, meta-analysis and meta-regression of the effect of protein supplementation on resistance training-induced gains in muscle mass and strength in healthy adults. Br J Sports Med. (2018) 52:376–84. 10.1136/bjsports-2017-097608 28698222 PMC5867436

[19] Grandjean A. Nutritional requirements to increase lean mass. Clin Sports Med. (1999) 18:623–32. 10.1016/S0278-5919(05)70172-1 10410845

[20] Houston ME. Gaining weight: the scientific basis of increasing skeletal muscle mass. Can J Appl Physiol. (1999) 24:305–16. 10.1139/h99-024 10470448

[21] Kreider RB. Dietary supplements and the promotion of muscle growth with resistance exercise. Sports Med. (1999) 27:97–110. 10.2165/00007256-199927020-00003 10091274

[22] Forbes GB Brown MR Welle SL Lipinski BA. Deliberate overfeeding in women and men: energy cost and composition of the weight gain. Br J Nutr. (1986) 56:1–9. 10.1079/BJN19860080 3479191

[23] Bray GA Smith SR de Jonge L Xie H Rood J Martin CK. Effect of dietary protein content on weight gain, energy expenditure, and body composition during overeating: a randomized controlled trial. JAMA. (2012) 307:47–55. 10.1001/jama.2011.1918 22215165 PMC3777747

[24] Churchward-Venne TA Murphy CH Longland TM Phillips SM. Role of protein and amino acids in promoting lean mass accretion with resistance exercise and attenuating lean mass loss during energy deficit in humans. Amino Acids. (2013) 45:231–40. 10.1007/s00726-013-1506-0 23645387

[25] Bray GA Redman LM de Jonge L Covington J Rood J Brock C. Effect of protein overfeeding on energy expenditure measured in a metabolic chamber. Am J Clin Nutr. (2015) 101:496–505. 10.3945/ajcn.114.091769 25733634

[26] Gorissen SH Burd NA Hamer HM Gijsen AP Groen BB van Loon LJ Carbohydrate coingestion delays dietary protein digestion and absorption but does not modulate postprandial muscle protein accretion. J Clin Endocrinol Metab. (2014) 99:2250–8. 10.1210/jc.2013-3970 24628553

[27] Koopman R Beelen M Stellingwerff T Pennings B Saris WH Kies AK Coingestion of carbohydrate with protein does not further augment postexercise muscle protein synthesis. Am J Physiol Endocrinol Metab. (2007) 293:E833–42. 10.1152/ajpendo.00135.2007 17609259

[28] Glynn EL Fry CS Timmerman KL Drummond MJ Volpi E Rasmussen BB Addition of carbohydrate or alanine to an essential amino acid mixture does not enhance human skeletal muscle protein anabolism. J Nutr. (2013) 143:307–14. 10.3945/jn.112.168203 23343676 PMC3713020

[29] Manore M Meyer N Thompson J Achieving healthy body weight. In: Sport Nutiriton for Health and Performance. 2nd ed Champaogn, IL: Human Kinetics (2009). p. 167–204.

[30] Williams MH Anderson DE Rawson ES Weight gaining through proper nutrition and exercise. In: Nutrition for Health, Fitness and Sport. 10th ed New York, NY: McGraw Hill (2013). p. 517–44.

[31] Moore D Phillips S Slater G Protein. In: Burke L Deakin V editors. Clinical Sports Nutrition. 5th ed North Ryde, NSW: McGRaw Hill (2015). p. 94–113.

[32] Dunford M Macedonio MA Weight management. In: Karpinski C Rosenbloom CA, editors. Sports Nutrition. A Handbook for Professionals. Chicago, IL: Academy of Nutrition and Dietetics (2018). p. 218–37.

[33] Josse AR Tang JE Tarnopolsky MA Phillips SM. Body composition and strength changes in women with milk and resistance exercise. Med Sci Sports Exerc. (2010) 42:1122–30. 10.1249/MSS.0b013e3181c854f6 19997019

[34] Longland TM Oikawa SY Mitchell CJ Devries MC Phillips SM. Higher compared with lower dietary protein during an energy deficit combined with intense exercise promotes greater lean mass gain and fat mass loss: a randomized trial. Am J Clin Nutr. (2016) 103:738–46. 10.3945/ajcn.115.119339 26817506

[35] Zemski AJ Keating SE Broad EM Marsh DJ Hind K Slater GJ. Preseason body composition adaptations in elite white and polynesian rugby union athletes. Int J Sport Nutr Exerc Metab. (2018) 29, 9–17. 10.1123/ijsnem.2018-0059 29757054

[36] Haakonssen EC Martin DT Burke LM Jenkins DG. Increased lean mass with reduced fat mass in an elite female cyclist returning to competition: case study. Int J Sports Physiol Perform. (2013) 8:699–701. 10.1123/ijspp.8.6.699 23538431

[37] Milsom J Barreira P Burgess DJ Iqbal Z Morton JP. Case study: muscle atrophy and hypertrophy in a premier league soccer player during rehabilitation from ACL injury. Int J Sport Nutr Exerc Metab. (2014) 24:543–52. 10.1123/ijsnem.2013-0209 24458224

[38] Jones B Till K Roe G O'Hara J Lees M Barlow MJ. Six-year body composition change in male elite senior rugby league players. J Sports Sci. (2018) 36:266–71. 10.1080/02640414.2017.1300313 28281879

[39] Elia M Stubbs RJ Henry CJ. Differences in fat, carbohydrate, and protein metabolism between lean and obese subjects undergoing total starvation. Obes Res. (1999) 7:597–604. 10.1002/j.1550-8528.1999.tb00720.x 10574520

[40] Bouchard C Tchernof A Tremblay A. Predictors of body composition and body energy changes in response to chronic overfeeding. Int J Obes. (2014) 38:236–42. 10.1038/ijo.2013.77 23736367 PMC3773296

[41] Till K Jones B Darrall-Jones J Emmonds S Cooke C. Longitudinal development of anthropometric and physical characteristics within academy rugby league players. J Strength Cond Res. (2015) 29:1713–22. 10.1519/JSC.0000000000000792 25474341

[42] Biolo G Tipton KD Klein S Wolfe RR. An abundant supply of amino acids enhances the metabolic effect of exercise on muscle protein. Am J Physiol. (1997) 273:E122–9. 10.1152/ajpendo.1997.273.1.E122 9252488

[43] Phillips SM Tipton KD Aarsland A Wolf SE Wolfe RR. Mixed muscle protein synthesis and breakdown after resistance exercise in humans. Am J Physiol. (1997) 273:E99–107. 10.1152/ajpendo.1997.273.1.E99 9252485

[44] Burd NA West DW Moore DR Atherton PJ Staples AW Prior T. Enhanced amino acid sensitivity of myofibrillar protein synthesis persists for up to 24 h after resistance exercise in young men. J Nutr. (2011) 141:568–73. 10.3945/jn.110.135038 21289204

[45] Bouchard C Tremblay A Despres JP Nadeau A Lupien PJ Theriault G. The response to long-term overfeeding in identical twins. N Engl J Med. (1990) 322:1477–82. 10.1056/NEJM199005243222101 2336074

[46] Leaf A Antonio J. The effects of overfeeding on body composition: the role of macronutrient composition - a narrative review. Int J Exerc Sci. (2017) 10:1275–96. 29399253 10.70252/HPPF5281 PMC5786199

[47] Goldberg AL Etlinger JD Goldspink DF Jablecki C. Mechanism of work-induced hypertrophy of skeletal muscle. Med Sci Sports. (1975) 7:185–98. 10.1249/00005768-197500730-00016 128681

[48] Gonzalez AM Hoffman JR Stout JR Fukuda DH Willoughby DS. Intramuscular anabolic signaling and endocrine response following resistance exercise: implications for muscle hypertrophy. Sports Med. (2016) 46:671–85. 10.1007/s40279-015-0450-4 26666743

[49] Wackerhage H Schoenfeld BJ Hamilton DL Lehti M Hulmi JJ. Stimuli and sensors that initiate skeletal muscle hypertrophy following resistance exercise. J Appl Physiol. (2019) 126:30–43. 10.1152/japplphysiol.00685.2018 30335577

[50] Hackett DA Johnson NA Chow CM. Training practices and ergogenic aids used by male bodybuilders. J Strength Cond Res. (2013) 27:1609–17. 10.1519/JSC.0b013e318271272a 22990567

[51] Kreider RB Kalman DS Antonio J Ziegenfuss TN Wildman R Collins R. International Society of Sports Nutrition position stand: safety and efficacy of creatine supplementation in exercise, sport, and medicine. J Int Soc Sports Nutr. (2017) 14:18. 10.1186/s12970-017-0173-z 28615996 PMC5469049

[52] Bier DM The energy costs of protein metabolism: lean and mean on uncle Sam's team. In: Poos MI Costello R Carlson-Newberry SJ, editors. The Role of Protein and Amino Acids in Sustaining and Enhancing Performance. Washington, DC: National Acadamies Press (1999). p. 109–19.

[53] Pasiakos SM Vislocky LM Carbone JW Altieri N Konopelski K Freake HC. Acute energy deprivation affects skeletal muscle protein synthesis and associated intracellular signaling proteins in physically active adults. J Nutr. (2010) 140:745–51. 10.3945/jn.109.118372 20164371

[54] Areta JL Burke LM Camera DM West DW Crawshay S Moore DR. Reduced resting skeletal muscle protein synthesis is rescued by resistance exercise and protein ingestion following short-term energy deficit. Am J Physiol Endocrinol Metab. (2014) 306:E989–97. 10.1152/ajpendo.00590.2013 24595305

[55] Donnelly JE Sharp T Houmard J Carlson MG Hill JO Whatley JE. Muscle hypertrophy with large-scale weight loss and resistance training. Am J Clin Nutr. (1993) 58:561–5. 10.1093/ajcn/58.4.561 8379514

[56] Nindl BC Harman EA Marx JO Gotshalk LA Frykman PN Lammi E. Regional body composition changes in women after 6 months of periodized physical training. J Appl Physiol. (2000) 88:2251–9. 10.1152/jappl.2000.88.6.2251 10846043

[57] Rozenek R Ward P Long S Garhammer J. Effects of high-calorie supplements on body composition and muscular strength following resistance training. J Sports Med Phys Fitness. (2002) 42:340–7. 12094125

[58] Rinehardt KF Effects of diet on muscle and strength gains during resistive training. In: Garrett WE Malone TR, editors. Ross Symposium on Muscle Development: Nutritional Alternatives to Anabolic Steroids. Columbus, OH: Ross (1988). p. 78–83.

[59] Ribeiro AS Nunes JP Schoenfeld BJ Aguiar AF Cyrino ES Effects of different dietary energy intake following resistance training on muscle mass and body fat in bodybuilders: a pilot study. J Hum Kinet. (2019). [Epub ahead of print]. 10.2478/hukin-2019-0038 PMC6942464 31915482

[60] Hamilton DL Philp A Can AMPK mediated suppression of mTORC1 explain the concurrent training effect? Cell Mol Exerc Physiol. (2013) 2:e4 10.7457/cmep.v2i1.e4

[61] Joosen AM Westerterp KR. Energy expenditure during overfeeding. Nutr Metab. (2006) 3:25. 10.1186/1743-7075-3-25 16836744 PMC1543621

[62] Forbes Dagger GB. Some adventures in body composition, with special reference to nutrition. Acta Diabetol. (2003) 40(suppl 1):S238–41. 10.1007/s00592-003-0075-1 14618482

[63] Spady DW Payne PR Picou D Waterlow JC. Energy balance during recovery from malnutrition. Am J Clin Nutr. (1976) 29:1073–88. 10.1093/ajcn/29.10.1073 823814

[64] Stock MJ. Gluttony and thermogenesis revisited. Int J Obes Relat Metab Disord. (1999) 23:1105–17. 10.1038/sj.ijo.0801108 10578199

[65] Chow CC Hall KD. The dynamics of human body weight change. PLoS Comput Biol. (2008) 4:e1000045. 10.1371/journal.pcbi.1000045 18369435 PMC2266991

[66] Thomas DM Martin CK Heymsfield S Redman LM Schoeller DA Levine JA. A simple model predicting individual weight change in humans. J Biol Dyn. (2011) 5:579–99. 10.1080/17513758.2010.508541 24707319 PMC3975626

[67] Scott CB Leighton BH Ahearn KJ McManus JJ. Aerobic, anaerobic, and excess postexercise oxygen consumption energy expenditure of muscular endurance and strength: 1-set of bench press to muscular fatigue. J Strength Cond Res. (2011) 25:903–8. 10.1519/JSC.0b013e3181c6a128 20703175

[68] Mookerjee S Welikonich MJ Ratamess NA. Comparison of energy expenditure during single-set vs. multiple-set resistance exercise. J Strength Cond Res. (2016) 30:1447–52. 10.1519/JSC.0000000000001230 26466137

[69] Roberson KB Jacobs KA White MJ Signorile JF. Loads and movement speed affect energy expenditure during circuit resistance exercise. Appl Physiol Nutr Metab. (2017) 42:637–46. 10.1139/apnm-2016-0552 28177703

[70] Rawson ES Walsh TM. Estimation of resistance exercise energy expenditure using accelerometry. Med Sci Sports Exerc. (2010) 42:622–8. 10.1249/MSS.0b013e3181b64ef3 19952824

[71] Stec MJ Rawson ES. Estimation of resistance exercise energy expenditure using triaxial accelerometry. J Strength Cond Res. (2012) 26:1413–22. 10.1519/JSC.0b013e318248d7b4 22222328

[72] Lytle JR Kravits DM Martin SE Green JS Crouse SF Lambert BS. Predicting energy expenditure of an acute resistance exercise. Med Sci Sports Exerc. (2019) 51:1532–7. 10.1249/MSS.0000000000001925 30768553

[73] Proud CG. Regulation of mammalian translation factors by nutrients. Eur J Biochem. (2002) 269:5338–49. 10.1046/j.1432-1033.2002.03292.x 12423332

[74] Browne GJ Proud CG. Regulation of peptide-chain elongation in mammalian cells. Eur J Biochem. (2002) 269:5360–8. 10.1046/j.1432-1033.2002.03290.x 12423334

[75] Waterlow JC. Protein turnover with special reference to man. Q J Exp Physiol. (1984) 69:409–38. 10.1113/expphysiol.1984.sp002829 6382379

[76] Elia M Organ and tissue contribution to metabolic rate. In: Kinney JM Tucker HN, editors. Energy Metabolism: Tissue Determinants and Cellular Corollaries. New York, NY: Raven (1992). p. 61–80.

[77] Illner K Brinkmann G Heller M Bosy-Westphal A Muller MJ. Metabolically active components of fat free mass and resting energy expenditure in nonobese adults. Am J Physiol Endocrinol Metab. (2000) 278:E308–15. 10.1152/ajpendo.2000.278.2.E308 10662716

[78] MacKenzie-Shalders KL Byrne NM King NA Slater GJ. Are increases in skeletal muscle mass accompanied by changes to resting metabolic rate in rugby athletes over a pre-season training period? Eur J Sport Sci. (2019) 19:885–92. 10.1080/17461391.2018.1561951 30614386

[79] Muller MJ Enderle J Pourhassan M Braun W Eggeling B Lagerpusch M. Metabolic adaptation to caloric restriction and subsequent refeeding: the Minnesota Starvation Experiment revisited. Am J Clin Nutr. (2015) 102:807–19. 10.3945/ajcn.115.109173 26399868

[80] Muller MJ Enderle J Bosy-Westphal A. Changes in energy expenditure with weight gain and weight loss in humans. Curr Obes Rep. (2016) 5:413–23. 10.1007/s13679-016-0237-4 27739007 PMC5097076

[81] Raben A Agerholm-Larsen L Flint A Holst JJ Astrup A. Meals with similar energy densities but rich in protein, fat, carbohydrate, or alcohol have different effects on energy expenditure and substrate metabolism but not on appetite and energy intake. Am J Clin Nutr. (2003) 77:91–100. 10.1093/ajcn/77.1.91 12499328

[82] Quatela A Callister R Patterson A MacDonald-Wicks L. The energy content and composition of meals consumed after an overnight fast and their effects on diet induced thermogenesis: a systematic review, meta-analyses and meta-regressions. Nutrients. (2016) 8:E670. 10.3390/nu8110670 27792142 PMC5133058

[83] Levine JA. Nonexercise activity thermogenesis (NEAT): environment and biology. Am J Physiol Endocrinol Metab. (2004) 286:E675–85. 10.1152/ajpendo.00562.2003 15102614

[84] Levine JA Eberhardt NL Jensen MD. Role of nonexercise activity thermogenesis in resistance to fat gain in humans. Science. (1999) 283:212–4. 10.1126/science.283.5399.212 9880251

[85] Mountjoy M Sundgot-Borgen J Burke L Carter S Constantini N Lebrun C. The IOC consensus statement: beyond the Female Athlete Triad–Relative Energy Deficiency in Sport (RED-S). Br J Sports Med. (2014) 48:491–7. 10.1136/bjsports-2014-093502 24620037

[86] Forbes GB Brown MR Welle SL Underwood LE. Hormonal response to overfeeding. Am J Clin Nutr. (1989) 49:608–11. 10.1093/ajcn/49.4.608 2648795

[87] Pritchard J Despres JP Gagnon J Tchernof A Nadeau A Tremblay A. Plasma adrenal, gonadal, and conjugated steroids before and after long-term overfeeding in identical twins. J Clin Endocrinol Metab. (1998) 83:3277–84. 10.1210/jc.83.9.3277 9745441

[88] Sato K Samocha-Bonet D Handelsman DJ Fujita S Wittert GA Heilbronn LK. Serum sex steroids and steroidogenesis-related enzyme expression in skeletal muscle during experimental weight gain in men. Diabetes Metab. (2014) 40:439–44. 10.1016/j.diabet.2014.03.006 24792219

[89] West DW Phillips SM. Associations of exercise-induced hormone profiles and gains in strength and hypertrophy in a large cohort after weight training. Eur J Appl Physiol. (2012) 112:2693–702. 10.1007/s00421-011-2246-z 22105707 PMC3371329

[90] Morton RW Sato K Gallaugher MPB Oikawa SY McNicholas PD Fujita S Muscle androgen receptor content but not systemic hormones is associated with resistance training-induced skeletal muscle hypertrophy in healthy, young men. Front Physiol. (2018) 9:1373 10.3389/fphys.2018.01373 30356739 PMC6189473

[91] MacKenzie-Shalders KL King NA Byrne NM Slater GJ. Increasing protein distribution has no effect on changes in lean mass during a rugby preseason. Int J Sport Nutr Exerc Metab. (2016) 26:1–7. 10.1123/ijsnem.2015-0040 26132746

[92] Stokes T Hector AJ Morton RW McGlory C Phillips SM. Recent perspectives regarding the role of dietary protein for the promotion of muscle hypertrophy with resistance exercise training. Nutrients. (2018) 10:E180. 10.3390/nu10020180 29414855 PMC5852756

[93] Antonio J Peacock CA Ellerbroek A Fromhoff B Silver T. The effects of consuming a high protein diet (4.4 g/kg/d) on body composition in resistance-trained individuals. J Int Soc Sports Nutr. (2014) 11:19. 10.1186/1550-2783-11-19 24834017 PMC4022420

[94] Antonio J Ellerbroek A Silver T Orris S Scheiner M Gonzalez A. A high protein diet (3.4 g/kg/d) combined with a heavy resistance training program improves body composition in healthy trained men and women–a follow-up investigation. J Int Soc Sports Nutr. (2015) 12:39. 10.1186/s12970-015-0100-0 26500462 PMC4617900

[95] Antonio J Ellerbroek A Silver T Vargas L Peacock C. The effects of a high protein diet on indices of health and body composition–a crossover trial in resistance-trained men. J Int Soc Sports Nutr. (2016) 13:3. 10.1186/s12970-016-0114-2 26778925 PMC4715299

[96] Halton TL Hu FB. The effects of high protein diets on thermogenesis, satiety and weight loss: a critical review. J Am Coll Nutr. (2004) 23:373–85. 10.1080/07315724.2004.10719381 15466943

[97] Eisenstein J Roberts SB Dallal G Saltzman E. High-protein weight-loss diets: are they safe and do they work? A review of the experimental and epidemiologic data. Nutr Rev. (2002) 60:189–200. 10.1301/00296640260184264 12144197

[98] Campbell BI Aguilar D Conlin L Vargas A Schoenfeld BJ Corson A. Effects of high versus low protein intake on body composition and maximal strength in aspiring female physique athletes engaging in an 8-week resistance training program. Int J Sport Nutr Exerc Metab. (2018) 28:580–5. 10.1123/ijsnem.2017-0389 29405780

[99] Devries MC Sithamparapillai A Brimble KS Banfield L Morton RW Phillips SM. Changes in kidney function do not differ between healthy adults consuming higher- compared with lower- or normal-protein diets: a systematic review and meta-analysis. J Nutr. (2018) 148:1760–75. 10.1093/jn/nxy197 30383278 PMC6236074

[100] Antonio J Ellerbroek A Silver T Vargas L Tamayo A Buehn R. A high protein diet has no harmful effects: a one-year crossover study in resistance-trained males. J Nutr Metab. (2016) 2016:9104792. 10.1155/2016/9104792 27807480 PMC5078648

[101] Horton TJ Drougas H Brachey A Reed GW Peters JC Hill JO. Fat and carbohydrate overfeeding in humans: different effects on energy storage. Am J Clin Nutr. (1995) 62:19–29. 10.1093/ajcn/62.1.19 7598063

[102] Lammert O Grunnet N Faber P Bjornsbo KS Dich J Larsen LO Effects of isoenergetic overfeeding of either carbohydrate or fat in young men. Br J Nutr. (2000) 84:233–45. 10.1017/S0007114500001471 11029975

[103] Tesch PA Colliander EB Kaiser P. Muscle metabolism during intense, heavy-resistance exercise. Eur J Appl Physiol Occup Physiol. (1986) 55:362–6. 10.1007/BF00422734 3758035

[104] Koopman R Manders RJ Jonkers RA Hul GB Kuipers H van Loon LJ. Intramyocellular lipid and glycogen content are reduced following resistance exercise in untrained healthy males. Eur J Appl Physiol. (2006) 96:525–34. 10.1007/s00421-005-0118-0 16369816

[105] Lambert CP Flynn MG Boone JBJ Michaud TJ Rodriguez-Zayas J Effects of carbohydrate feeding on multiple-bout resistance exercise. J Strength Cond Res. (1991) 5:192–7. 10.1519/00124278-199111000-00004

[106] Haff GG Schroeder CA Koch AJ Kuphal KE Comeau MJ Potteiger JA. The effects of supplemental carbohydrate ingestion on intermittent isokinetic leg exercise. J Sports Med Phys Fitness. (2001) 41:216–22. 11447365

[107] Tesch PA Ploutz-Snyder LL Ystrom L Castro MJ Dudley GA Skeletal muscle glycogen loss evoked by resistance exercise. J Strength Cond Res. (1998) 12:67–73. 10.1519/00124278-199805000-00001

[108] Mitchell JB DiLauro PC Pizza FX Cavender DL. The effect of preexercise carbohydrate status on resistance exercise performance. Int J Sport Nutr. (1997) 7:185–96. 10.1123/ijsn.7.3.185 9286742

[109] Greene DA Varley BJ Hartwig TB Chapman P Rigney M. A low-carbohydrate ketogenic diet reduces body mass without compromising performance in powerlifting and olympic weightlifting athletes. J Strength Cond Res. (2018) 32:3373–82. 10.1519/JSC.0000000000002904 30335720

[110] Kephart WC Pledge CD Roberson PA Mumford PW Romero MA Mobley CB. The three-month effects of a ketogenic diet on body composition, blood parameters, and performance metrics in crossfit trainees: a pilot study. Sports. (2018) 6:1. 10.3390/sports6010001 29910305 PMC5969192

[111] Vargas S Romance R Petro JL Bonilla DA Galancho I Espinar S. Efficacy of ketogenic diet on body composition during resistance training in trained men: a randomized controlled trial. J Int Soc Sports Nutr. (2018) 15:31. 10.1186/s12970-018-0236-9 29986720 PMC6038311

[112] Slater G Phillips SM. Nutrition guidelines for strength sports: sprinting, weightlifting, throwing events, and bodybuilding. J Sports Sci. (2011) 29 (suppl 1):S67–77. 10.1080/02640414.2011.574722 21660839

[113] Thomas DT Erdman KA Burke LM. American college of sports medicine joint position statement. Nutrition and athletic performance. Med Sci Sports Exerc. (2016) 48:543–68. 10.1249/MSS.0000000000000852 26891166

[114] Jequier E. Response to and range of acceptable fat intake in adults. Eur J Clin Nutr. (1999) 53(suppl 1):S84–8. 10.1038/sj.ejcn.1600747 10365984

[115] Trumbo P Schlicker S Yates AA Poos M. Dietary reference intakes for energy, carbohydrate, fiber, fat, fatty acids, cholesterol, protein and amino acids. J Am Diet Assoc. (2002) 102:1621–30. 10.1016/S0002-8223(02)90346-9 12449285

[116] Wang C Catlin DH Starcevic B Heber D Ambler C Berman N. Low-fat high-fiber diet decreased serum and urine androgens in men. J Clin Endocrinol Metab. (2005) 90:3550–9. 10.1210/jc.2004-1530 15741266

[117] Hamalainen E Adlercreutz H Puska P Pietinen P. Diet and serum sex hormones in healthy men. J Steroid Biochem. (1984) 20:459–64. 10.1016/0022-4731(84)90254-1 6538617

[118] Dorgan JF Judd JT Longcope C Brown C Schatzkin A Clevidence BA. Effects of dietary fat and fiber on plasma and urine androgens and estrogens in men: a controlled feeding study. Am J Clin Nutr. (1996) 64:850–5. 10.1093/ajcn/64.6.850 8942407

[119] Collins CE O'Loughlin EV Henry RL. Fat gram target to achieve high energy intake in cystic fibrosis. J Paediatr Child Health. (1997) 33:142–7. 10.1111/j.1440-1754.1997.tb01017.x 9145358

[120] Rosqvist F Iggman D Kullberg J Cedernaes J Johansson HE Larsson A. Overfeeding polyunsaturated and saturated fat causes distinct effects on liver and visceral fat accumulation in humans. Diabetes. (2014) 63:2356–68. 10.2337/db13-1622 24550191

[121] Smith GI Atherton P Reeds DN Mohammed BS Rankin D Rennie MJ. Omega-3 polyunsaturated fatty acids augment the muscle protein anabolic response to hyperinsulinaemia-hyperaminoacidaemia in healthy young and middle-aged men and women. Clin Sci. (2011) 121:267–78. 10.1042/CS20100597 21501117 PMC3499967

[122] Smith GI Julliand S Reeds DN Sinacore DR Klein S Mittendorfer B. Fish oil-derived n-3 PUFA therapy increases muscle mass and function in healthy older adults. Am J Clin Nutr. (2015) 102:115–22. 10.3945/ajcn.114.105833 25994567 PMC4480667

[123] Piers LS Walker KZ Stoney RM Soares MJ O'Dea K. The influence of the type of dietary fat on postprandial fat oxidation rates: monounsaturated (olive oil) vs saturated fat (cream). Int J Obes Relat Metab Disord. (2002) 26:814–21. 10.1038/sj.ijo.0801993 12037652

[124] Piers LS Walker KZ Stoney RM Soares MJ O'Dea K. Substitution of saturated with monounsaturated fat in a 4-week diet affects body weight and composition of overweight and obese men. Br J Nutr. (2003) 90:717–27. 10.1079/BJN2003948 13129479

[125] Tang JE Moore DR Kujbida GW Tarnopolsky MA Phillips SM. Ingestion of whey hydrolysate, casein, or soy protein isolate: effects on mixed muscle protein synthesis at rest and following resistance exercise in young men. J Appl Physiol. (2009) 107:987–92. 10.1152/japplphysiol.00076.2009 19589961

[126] van Vliet S Shy EL Abou Sawan S Beals JW West DW Skinner SK. Consumption of whole eggs promotes greater stimulation of postexercise muscle protein synthesis than consumption of isonitrogenous amounts of egg whites in young men. Am J Clin Nutr. (2017) 106:1401–12. 10.3945/ajcn.117.159855 28978542

[127] Elliot TA Cree MG Sanford AP Wolfe RR Tipton KD. Milk ingestion stimulates net muscle protein synthesis following resistance exercise. Med Sci Sports Exerc. (2006) 38:667–74. 10.1249/01.mss.0000210190.64458.25 16679981

[128] Burd NA Beals JW Martinez IG Salvador AF Skinner SK. Food-first approach to enhance the regulation of post-exercise skeletal muscle protein synthesis and remodeling. Sports Med. (2019) 49:59–68. 10.1007/s40279-018-1009-y 30671904 PMC6445816

[129] Kerksick CM Arent S Schoenfeld BJ Stout JR Campbell B Wilborn CD. International society of sports nutrition position stand: nutrient timing. J Int Soc Sports Nutr. (2017) 14:33. 10.1186/s12970-017-0189-4 28919842 PMC5596471

[130] Alghannam AF Gonzalez JT Betts JA. Restoration of muscle glycogen and functional capacity: role of post-exercise carbohydrate and protein co-ingestion. Nutrients. (2018) 10:E253. 10.3390/nu10020253 29473893 PMC5852829

[131] Krings BM Rountree JA McAllister MJ Cummings PM Peterson TJ Fountain BJ. Effects of acute carbohydrate ingestion on anaerobic exercise performance. J Int Soc Sports Nutr. (2016) 13:40. 10.1186/s12970-016-0152-9 27843418 PMC5105234

[132] Kulik JR Touchberry CD Kawamori N Blumert PA Crum AJ Haff GG Supplemental carbohydrate ingestion does not improve performance of high-intensity resistance exercise. J Strength Cond Res. (2008) 22:1101–7. 10.1519/JSC.0b013e31816d679b 18545201

[133] Escobar KA Morales J Vandusseldorp TA. The effect of a moderately low and high carbohydrate intake on crossfit performance. Int J Exerc Sci. (2016) 9:460–70. 27766133 10.70252/IZLO1608 PMC5065325

[134] Rountree JA Krings BM Peterson TJ Thigpen AG McAllister MJ Holmes ME. Efficacy of carbohydrate ingestion on crossfit exercise performance. Sports. (2017) 5:61. 10.3390/sports5030061 29910421 PMC5968949

[135] Phillips SM Van Loon LJ. Dietary protein for athletes: from requirements to optimum adaptation. J Sports Sci. (2011) 29(suppl 1):S29–38. 10.1080/02640414.2011.619204 22150425

[136] Atherton PJ Smith K. Muscle protein synthesis in response to nutrition and exercise. J Physiol. (2012) 590:1049–57. 10.1113/jphysiol.2011.225003 22289911 PMC3381813

[137] Dashti HS Mogensen KM. Recommending small, frequent meals in the clinical care of adults: a review of the evidence and important considerations. Nutr Clin Pract. (2017) 32:365–77. 10.1177/0884533616662995 27589258

[138] Perrigue MM Drewnowski A Wang CY Neuhouser ML Higher eating frequency does not decrease appetite in healthy adults. J Nutr. (2016) 146:59–64. 10.3945/jn.115.216978 26561409 PMC4700979

[139] Burke LM Slater G Broad EM Haukka J Modulon S Hopkins WG. Eating patterns and meal frequency of elite Australian athletes. Int J Sport Nutr Exerc Metab. (2003) 13:521–38. 10.1123/ijsnem.13.4.521 14967874

[140] Erdman KA Tunnicliffe J Lun VM Reimer RA. Eating patterns and composition of meals and snacks in elite Canadian athletes. Int J Sport Nutr Exerc Metab. (2013) 23:210–9. 10.1123/ijsnem.23.3.210 23114732

[141] de Castro JM Bellisle F Feunekes GIJ Dalix A-M De Graaf C Culture and meal patterns: a comparison of the food intake of free-living American, Dutch, and French students. Nutr Res. (1997) 17:807–29. 10.1016/S0271-5317(97)00050-X

[142] Anderson L Naughton RJ Close GL Di Michele R Morgans R Drust B. Daily distribution of macronutrient intakes of professional soccer players from the english premier league. Int J Sport Nutr Exerc Metab. (2017) 27:491–8. 10.1123/ijsnem.2016-0265 28657805

[143] Deutz RC Benardot D Martin DE Cody MM. Relationship between energy deficits and body composition in elite female gymnasts and runners. Med Sci Sports Exerc. (2000) 32:659–68. 10.1097/00005768-200003000-00017 10731010

[144] Fahrenholtz IL Sjodin A Benardot D Tornberg AB Skouby S Faber J. Within-day energy deficiency and reproductive function in female endurance athletes. Scand J Med Sci Sports. (2018) 28:1139–46. 10.1111/sms.13030 29205517

[145] Torstveit MK Fahrenholtz I Stenqvist TB Sylta O Melin A. Within-day energy deficiency and metabolic perturbation in male endurance athletes. Int J Sport Nutr Exerc Metab. (2018) 28:419–27. 10.1123/ijsnem.2017-0337 29405793

[146] Cribb PJ Hayes A. Effects of supplement timing and resistance exercise on skeletal muscle hypertrophy. Med Sci Sports Exerc. (2006) 38:1918–25. 10.1249/01.mss.0000233790.08788.3e 17095924

[147] Hoffman JR Ratamess NA Tranchina CP Rashti SL Kang J Faigenbaum AD. Effect of protein-supplement timing on strength, power, and body-composition changes in resistance-trained men. Int J Sport Nutr Exerc Metab. (2009) 19:172–85. 10.1123/ijsnem.19.2.172 19478342

[148] Bo S Musso G Beccuti G Fadda M Fedele D Gambino R. Consuming more of daily caloric intake at dinner predisposes to obesity. A 6-year population-based prospective cohort study. PLoS ONE. (2014) 9:e108467. 10.1371/journal.pone.0108467 25250617 PMC4177396

[149] Wang JB Patterson RE Ang A Emond JA Shetty N Arab L. Timing of energy intake during the day is associated with the risk of obesity in adults. J Hum Nutr Diet. (2014) 27(suppl 2):255–62. 10.1111/jhn.12141 23808897

[150] Allison KC Goel N. Timing of eating in adults across the weight spectrum: metabolic factors and potential circadian mechanisms. Physiol Behav. (2018) 192:158–66. 10.1016/j.physbeh.2018.02.047 29486170 PMC6019166

[151] Timlin MT Pereira MA. Breakfast frequency and quality in the etiology of adult obesity and chronic diseases. Nutr Rev. (2007) 65:268–81. 10.1111/j.1753-4887.2007.tb00304.x 17605303

[152] de Castro JM. The time of day of food intake influences overall intake in humans. J Nutr. (2004) 134:104–11. 10.1093/jn/134.1.104 14704301

[153] Snijders T Res PT Smeets JS van Vliet S van Kranenburg J Maase K. Protein ingestion before sleep increases muscle mass and strength gains during prolonged resistance-type exercise training in healthy young men. J Nutr. (2015) 145:1178–84. 10.3945/jn.114.208371 25926415

[154] Bradley WJ Cavanagh B Douglas W Donovan TF Twist C Morton JP. Energy intake and expenditure assessed ‘in-season’ in an elite European rugby union squad. Eur J Sport Sci. (2015) 15:469–79. 10.1080/17461391.2015.1042528 26055695

[155] King NA Caudwell PP Hopkins M Stubbs JR Naslund E Blundell JE. Dual-process action of exercise on appetite control: increase in orexigenic drive but improvement in meal-induced satiety. Am J Clin Nutr. (2009) 90:921–7. 10.3945/ajcn.2009.27706 19675105

[156] Peos JJ Norton LE Helms ER Galpin AJ Fournier P. Intermittent dieting: theoretical considerations for the athlete. Sports. (2019) 7:E22. 10.3390/sports7010022 30654501 PMC6359485

[157] Sagayama H Jikumaru Y Hirata A Yamada Y Yoshimura E Ichikawa M. Measurement of body composition in response to a short period of overfeeding. J Physiol Anthropol. (2014) 33:29. 10.1186/1880-6805-33-29 25208693 PMC4237876

[158] DellaValle DM Roe LS Rolls BJ. Does the consumption of caloric and non-caloric beverages with a meal affect energy intake? Appetite. (2005) 44:187–93. 10.1016/j.appet.2004.11.003 15808893

[159] Mourao DM Bressan J Campbell WW Mattes RD. Effects of food form on appetite and energy intake in lean and obese young adults. Int J Obes. (2007) 31:1688–95. 10.1038/sj.ijo.0803667 17579632

[160] Rolls BJ Roe LS Meengs JS. Salad and satiety: energy density and portion size of a first-course salad affect energy intake at lunch. J Am Diet Assoc. (2004) 104:1570–6. 10.1016/j.jada.2004.07.001 15389416

[161] Aune D Giovannucci E Boffetta P Fadnes LT Keum N Norat T. Fruit and vegetable intake and the risk of cardiovascular disease, total cancer and all-cause mortality-a systematic review and dose-response meta-analysis of prospective studies. Int J Epidemiol. (2017) 46:1029–56. 10.1093/ije/dyw319 28338764 PMC5837313

[162] Westerterp-Plantenga MS Lemmens SG Westerterp KR. Dietary protein - its role in satiety, energetics, weight loss and health. Br J Nutr. (2012) 108(suppl 2):S105–12. 10.1017/S0007114512002589 23107521

[163] Bone JL Ross ML Tomcik KA Jeacocke NA Hopkins WG Burke LM. Manipulation of muscle creatine and glycogen changes dual X-ray absorptiometry estimates of body composition. Med Sci Sports Exerc. (2017) 49:1029–35. 10.1249/MSS.0000000000001174 28410328

[164] Shiose K Yamada Y Motonaga K Sagayama H Higaki Y Tanaka H. Segmental extracellular and intracellular water distribution and muscle glycogen after 72-h carbohydrate loading using spectroscopic techniques. J Appl Physiol. (2016) 121:205–11. 10.1152/japplphysiol.00126.2016 27231310

[165] Kristiansen MS Uhrbrand A Hansen M Shiguetomi-Medina JM Vissing K Stodkilde-Jorgensen H. Concomitant changes in cross-sectional area and water content in skeletal muscle after resistance exercise. Scand J Med Sci Sports. (2014) 24:e260–8. 10.1111/sms.12160 24330190

[166] Garthe I Raastad T Refsnes PE Sundgot-Borgen J. Effect of nutritional intervention on body composition and performance in elite athletes. Eur J Sport Sci. (2013) 13:295–303. 10.1080/17461391.2011.643923 23679146

